# The Transcription Factor MdERF78 Is Involved in ALA-Induced Anthocyanin Accumulation in Apples

**DOI:** 10.3389/fpls.2022.915197

**Published:** 2022-06-02

**Authors:** Xiang Fang, Liuzi Zhang, Liangju Wang

**Affiliations:** College of Horticulture, Nanjing Agricultural University, Nanjing, China

**Keywords:** ALA, anthocyanin, apple, MdERF78, regulatory mechanism

## Abstract

As a friendly plant growth regulator to the environment, 5-aminolevulinic acid (ALA) has been widely used in plant production, such as fruit coloration, stress resistance, and so on. Previous studies have identified some genes that have a function in the anthocyanin accumulation induced by ALA. However, the regulatory mechanism has not been well revealed. In the current study, we proposed that an ALA-responsive transcription factor, MdERF78, regulated anthocyanin accumulation. *MdERF78*, overexpressed in apple peels or calli, resulted in a significant increase of anthocyanins, while *MdERF78* interference had an opposite trend. Furthermore, the anthocyanin accumulation induced by *MdERF78* overexpression was enhanced by exogenous ALA treatment, suggesting that MdERF78 was involved in the ALA-induced anthocyanin accumulation. Yeast one-hybrid and dual luciferase reporter assays revealed that MdERF78 bound to the promoters of *MdF3H* and *MdANS* directly and activated their expressions. Additionally, MdERF78 interacted with MdMYB1 and enhanced the transcriptional activity of MdMYB1 to its target gene promoters. Based on these, it can be concluded that MdERF78 has a positive function in ALA-induced anthocyanin accumulation via the MdERF78-*MdF3Hpro*/*MdANSpro* and MdERF78-MdMYB1-*MdDFRpro/MdUFGTpro/MdGSTF12pro* regulatory network. These findings provide new insights into the regulatory mechanism of ALA-promoted anthocyanin accumulation.

## Introduction

Fruits with red, blue, or purple skin and flesh usually have a higher market value because the bright color is more attractive to the consumers (Pathare et al., 2013). As a secondary metabolite, anthocyanins are widely distributed in plants and have diverse biological functions, including biotic and abiotic stress defenses (Lev-Yadun and Gould, 2008; Landi et al., 2015), pollinator attraction, and seed dispersers (Shang et al., 2011). In addition, anthocyanins are beneficial to human health, help to maintain cardiovascular fitness, and prevent oxidative stress (He and Giusti, 2010).

Anthocyanins are synthesized in the cytosol through a series of enzymes, including the early synthetic enzymes CHS (chalcone synthase), CHI (chalcone isomerase), and F3H (flavanone 3-hydroxylase F3H), and late biosynthetic enzymes DFR (dihydroflavonol 4-reductase), ANS (anthocyanidin synthase), and UFGT (UDP-glucose flavonoid 3-O-glucosyltransferase; Honda et al., 2002), eventually accumulating in the vacuole for storage. Glutathione S-transferase (GST) is one of the crucial proteins for anthocyanin transport (Zhao, 2015). The gene mutation or knockdown results in decreased fruit coloration in cultivated strawberries (Luo et al., 2018).

The structural genes involved in anthocyanin biosynthesis and transport pathway are regulated by a variety of transcription factors. MBW (R2R3-MYB, bHLH, and WD40) complex is known as the core regulator in anthocyanin accumulation (Allan et al., 2008; Xu et al., 2015). In apple, MdMYB1 (Takos et al., 2006), MdMYB10 (Espley et al., 2007), and MdMYBA (Ban et al., 2007), which are allelic to each other, play positive roles in anthocyanin biosynthesis. MdMYB3 (Vimolmangkang et al., 2013), MdMYB9, MdMYB11 (An et al., 2015), MdMYB90 (Sun et al., 2021), and MdMYB110a (Umemura et al., 2013) also promote anthocyanin accumulation. MdMYBA promotes anthocyanin synthesis by binding to the promoter of *MdANS* (Ban et al., 2007), while MdMYB1 activates the *MdGSTF6* expression to promote anthocyanin transport (Jiang et al., 2019). MdMYB114 directly binds to the promoters of *MdANS*, *MdUFGT*, and *MdGST* to enhance anthocyanin biosynthesis and transport independent of the MBW complex, and MdMYB114 expression itself is activated by MdbZIP4-like, a basic leucine-zipper TF by binding to the G-box of the gene promoter (Jiang et al., 2021). Therefore, MYBs can regulate anthocyanin biosynthesis and transport through multiple routes and at different levels, and the expressions themselves are in turn regulated by the other TFs. On the other hand, some other MYB TFs like MdMYB16 (Xu et al., 2017), MdMYB15L (Xu et al., 2018), MdMYB111 (Yang et al., 2019), and MdMYB6 (Xu et al., 2020) are known as negative regulators in anthocyanin biosynthesis. It makes the regulatory net more complicated. Furthermore, MdbHLH3 itself can promote the transcription of the key structural genes such as *MdDFR* and *MdUFGT*, and the activation can be enhanced by the binding of MdMYB1 (Xie et al., 2012). WD40, which is an important component of MBW usually interacts with bHLH (Ramsay and Glover, 2005), where MdbHLH3 or MdbHLH33 interact with MdTTG1 to form a complex (An et al., 2012). In addition, other transcription factors like MADS (Lalusin et al., 2006), bZIP (An et al., 2017), WRKY (Zhang et al., 2019), NAC (Zhang et al., 2020a), and ERF (Ma et al., 2021) are also reported to participate in the regulation of anthocyanin accumulation.

The accumulation of anthocyanin is affected by various physical and chemical factors, including temperature (Ubi, 2004), light (Jaakola, 2013), abiotic stress (e.g., drought and wounding stress; An et al., 2019a; An et al., 2020b), and plant hormones (e.g., ethephon and MeJA; Gao et al., 2021). As a key biosynthetic precursor of tetrapyrrole compounds, ALA is widely applied in agricultural production to improve plant growth and stress resistance (Hotta et al., 1997; Wang et al., 2003; Akram and Ashraf, 2013). In apples, ALA was firstly found to promote fruit coloration (Wang et al., 2004), and the effect has also been verified in pear (Xiao et al., 2012), litchi (Feng et al., 2015), peach (Ye et al., 2017), and grape (Zhang et al., 2021). It is believed that ALA promotes anthocyanin accumulation by upregulating transcripts of structural and regulatory genes involved in anthocyanin biosynthesis and transport pathways (Zhang et al., 2021; Zheng et al., 2021). MdMADS1 has been suggested to be a transcription factor to regulate the structural gene expressions during ALA-promoted anthocyanin accumulation in apple fruits and calli (Feng et al., 2016). MdMYB9 and MdMYB10 are also proposed to be involved in ALA-induced anthocyanin accumulation by directly activating the transcription of *MdMATE8* (Zheng et al., 2021). Nevertheless, the regulatory mechanisms of ALA in inducing anthocyanin accumulation need further study. Additionally, previous studies have shown that *ACO1*, a key ethylene synthesis gene, was also upregulated during the process of ALA-induced anthocyanin accumulation in apple calli (Feng et al., 2016). ALA and ethylene share similar functions in anthocyanin accumulation but no further study has been carried out on the relationship between ALA and ethylene.

Ethylene responsive factors (ERFs), which are important for plant growth, development, and resistance stresses have been identified in various plants (Xu et al., 2011; Mizoi et al., 2012). It is also reported that ERFs can be stimulated by abscisic acid (Wang et al., 2015), jasmonate (Yu et al., 2012), ethylene, and auxin (Liu et al., 2018). More and more evidence has shown that ERFs contribute to the regulation of anthocyanin biosynthesis. For instance, PyERF3 interacts with PyMYB114 to co-regulate anthocyanin biosynthesis in pears (Yao et al., 2017). Blue light induced the expression of *Pp4ERF24* and *Pp12ERF96*, which can interact with PpMYB114 to contribute to anthocyanin biosynthesis in red pear (Ni et al., 2019). In apple, *MdERF3* is directly activated by MdMYB1 resulting in the release of ethylene, which further induces the expression of *MdEIL1*. MdEIL1 activates the expression of *MdMYB1* by binding its promoter, thereby promoting anthocyanin accumulation (An et al., 2018a). MdERF1B not only interacts with MdMYB1, MdMYB9s, and MdMYB11 but also directly activates the expressions of *MdMYB9* and *MdMYB11* to improve ethylene-induced anthocyanin accumulation (Zhang et al., 2018). Recently, a MdWRKY1–MdLNC499–MdERF109 cascade is proposed to regulate light-promoted anthocyanin biosynthesis in apple fruit (Ma et al., 2021). However, whether ERFs are involved in ALA-induced anthocyanin accumulation is not clear.

In a previous study of our group (Zheng et al., 2021), the RNA-seq data have shown that many ERFs after ALA treatment fall in the differentially expressed genes (DEG) category. Nevertheless, their functions were not analyzed. In this study, we tested the effects of ethephon (Eth), ALA, and 1-MCP (1-methylcyclopropene), an ethylene inhibitor on apple fruit coloration and gene expressions, from which we observed an AP2/ERF TF, MdERF78 closely responsive to ALA and Eth treatments. Overexpression of *MdERF78* in apple fruits or calli induce significant accumulation of anthocyanins, which was further enhanced by exogenous ALA. Y1H (Yeast one-hybrid) and dual luciferase assay show that MdERF78 directly binds to the promoters of *MdF3H* and *MdANS* and activates their expressions. In addition, MdERF78 interacts with MdMYB1, a key positive regulator of anthocyanin accumulation in apple, and improves the transcriptional activity of MdMYB1 to its target gene promoters. Taken together, our studies provide a new idea on the molecular mechanisms of ALA-promoted anthocyanin accumulation.

## Materials and Methods

### Plant Materials and Treatments

Bagged apple (*Malus domestica* cv. “Gala”) fruits, which were harvested at 160 days after full bloom were treated with Eth, ALA, 1-MCP, ALA + Eth, and ALA + 1-MCP, respectively. For the Eth, ALA, and Eth + ALA treatments, the detached fruits were dipped in 300 mg L^–1^ Eth or 200 mg L^–1^ ALA solution or the mix of them for 1 min, where Tween-20 was added as an emulsifier. For the 1-MCP treatment, apples were fumigated in an air-tight container with 1 μL/L 1-MCP for 24 h. For the ALA + 1-MCP treatment, apples were pre-fumigated with 1-MCP and then treated with the ALA solution. Each treatment included 6 apples that treated with clear water as the control. All fruits were kept in darkness overnight, then transferred into a phytotron with constant light (200 μmol m^–2^ s^–1^) for 4 days at 18^°^C, and the peels were collected from four different sides of an apple for further use.

Apple (*Malus domestica* cv. “Orin”) calli were cultured according to An et al. (2017). For transgenic apple calli (see below), 0.17 mg L^–1^ ALA was added to the MS medium, then incubated in darkness for 3 days, and transferred to phytotron with constant light (200 μmol m^–2^s^–1^) at 18^°^C for coloring.

### Measurement of Anthocyanins

For the extraction and measurement of total anthocyanins of apples, we referred to Zheng et al. (2021). Plant tissues were ground with liquid nitrogen quickly and then extracted with 1 mL 1% (v/v) HCl methanol at 4^°^C for 24 h in darkness. The anthocyanins were determined by a multifunctional microplate reader (Bio-Tek, America).

### RNA Extraction and qRT-PCR Analysis

RNA Extraction Kit (TIANGEN, China) was used to extract total RNA from apple peels or calli. Reverse transcription was carried out by a PrimeScript™ RT reagent Kit (Transgen Biotech, China). 2 × SYBR Green qPCR Mix (Vazyme, China) and ABI QuantStudio 6Flex (Bio-Rad, United States) were used to measure the relative expressions of genes (Fang et al., 2020). The expressions were calculated with the 2^–ΔΔ*Ct*^ method (Livak and Schmitten, 2010), with three biological replications for each sample. All primes were listed in Supplementary Table 1.

### Sequence and Domain Analysis of MdERFs

The protein sequences used for phylogenetic analysis were obtained from the NCBI (National Center for Biotechnology Information) database. Sequence alignment was performed by DNAMAN. A phylogenetic tree comprising MdERF78 and other known anthocyanin-related ERFs was constructed by the MEGA7.0 program based on the neighbor-joining method.

### Subcellular Localization Analysis of MdERF78

The CDS of *MdERF78* excluding the terminal codon was cloned into pCAMBIA1302 vector to generate *35S:MdERF78:GFP* plasmid. Then, the recombinant vector and the empty ones were, respectively, transfected into *Agrobacterium tumefaciens* GV3101, transiently transformed into 4-week-old tobacco (*Nicotiana benthamiana*) leaves according to a previous method (Fan et al., 2016). After inoculation for 48 h, the enhanced GFP fluorescence signal was detected under a confocal microscopy with the excitation wavelength at 488 nm and emission wavelength at 490–550 nm (LSM 780, Zeiss, Germany).

### Fruit Skin Injection and Callus Genetic Transformation of Apple

The *35S*:*MdERF78:GFP* plasmid for subcellular localization analysis was used for the overexpression of MdERF78 in fruit skin injection. VIGS (virus-induced gene silencing) was used to silence the gene by inserting the *MdERF78* CDS into pTRV2 (tobacco rattle virus). The empty pCAMBIA1302 and TRV (TRV1 + TRV2) vectors were used as the controls. The method of apple skin injections was according to An et al. (2020b). For stable transformation in “Orin” apple calli, the same overexpression vector (*35S*:*MdERF78:GFP*) was used, similar to the transient genetic transformation. A 421bp gene fragment of *MdERF78* was inserted into RNAi vector (pHELLSGATE4) for silence expression. Two-week-old calli were infected with *Agrobacterium for* about 30 min and then cocultured on MS medium in darkness for 1–2 days at 24^°^C. Then they were evenly spread on MS medium with different antibiotics. The cell lines of *MdERF78* overexpression or silence were confirmed by PCR and qRT-PCR.

### Transcriptome Analysis

For transcriptome analysis, the overexpressed, interfering expressed *MdERF78* calli of apples and the wild type, which had been already exposed to light for 7 days, were used for RNA-seq, performed by Novogene (Beijing, China). The reads mapping and counting were performed by HISAT and HTSeq (Anders et al., 2015; Kim et al., 2015). The differentially expressed genes (DEGs) were identified as the genes with | log_2_(Fold Change) | > 1 and FDR ≤ 0.05. KOBAS (v2.052) and Blast2GO were used for the KEGG pathway and GO enrichment analysis of the DEGs (Conesa et al., 2005; Xie et al., 2011). The heat map was plotted with log_2_RPKM to show visually the differences in expressions (Chen et al., 2020).

### Yeast One-Hybrid Assay

The promoter fragments, about 2,000 bp of *MdCHS*, *MdCHI, MdF3H, MdDFR*, *MdANS, MdUFGT*, *MdGSTF12*, and *MdMYB1*, were fused into the pAbAi vector, respectively. The ERF78-pGADT7 vectors were transformed into competent cells of yeast containing the promoter sequences. The empty pGADT7 plasmid was used as the control. The SD/-Leu medium supplement with AbA (aureobasidin A, Takara, Japan) was used as he selection medium to observe whether MdERF78 binds to the downstream genes.

### Dual Luciferase Assays

To detect luciferase activities, the promoter fragments, about 2,000 bp of *MdF3H*, *MdDFR*, *MdANS*, *MdUFGT*, and *MdGSTF12*, were fused into pGreenII 0800-LUC plasmid as the reporter, while the CDS of *MdMYB1* and *MdERF78* were inserted into the pGreenII 62-SK plasmid as the effector. All of the above vectors were transfected into *Agrobacterium* GV3101-psoup, respectively and then injected into tobacco leaves with different combinations. The luciferase signals were observed by an imaging apparatus (PIXIS 1024B, United States). A luciferase detection Kit (Transgen, Beijing, China) was used to examine the transcriptional activity. The transcriptional abilities were expressed by the LUC/REN ratio.

### Yeast Two-Hybrid Assays

The CDS of *MdERF78* was inserted into the pGBKT7 construct as the bait, and the CDS of *MdMYB1* and *MdbHLH3* were inserted into the pGADT7 constructs as the prey. Bait and prey plasmids were co-transformed into yeast two-hybrid (Y2H) Gold cells mediated by PEG. The transformed yeast cells were grown on *SD*/-T/-L (-tryptophan/- leucine, DDO) medium for 3 days. Then the positive clones were dotted on the selection medium (-tryptophan/-leucine/-histidine/-adenine + X-α-gal + AbA, QDO) to observe yeast growth.

### Bimolecular Fluorescence Complementation Assays

For the bimolecular fluorescence complementation (BiFC) assay, we used two systems, i.e., onion epidermis and tobacco leaves. The CDS of *MdMYB1* and *MdERF78* genes were cloned into the plasmids YCE and YNE, respectively. Onion epidermis was infected with *Agrobacterium* solution which contained different combinations of plasmids for 30 min. Then the onion epidermis was placed on the MS solid medium in darkness for 48 h at room temperature. Meanwhile, tobacco leaves were injected with *Agrobacterium* infection solution and then kept at room temperature for 48 h. The YFP (yellow fluorescence protein) fluorescence in both onion and tobacco was detected under a confocal microscopy at the excitation wavelength 488 nm and emission wavelength at 510–565 nm (LSM 780, Zeiss, Germany).

### GUS Staining

The promoters, about 2,000 bp of *MdDFR*, *MdUFGT*, and *MdGSTF12*, were fused to pBI121-GUS to replace its 35S promoter used for the reporter vector. MdMYB1 and MdERF78 were fused into the vector driven by CaMV35S. The resultant constructs were transfected into *Agrobacterium* strain GV3101 and then injected into 4-weeks old tobacco leaves. After 3 days, the leaves were used for GUS staining. The method was carried out as previously described (Jefferson, 1987).

### Accession Numbers

The accession numbers are as follows: *MdERF78* (MD15G1036500), *MdPAL* (MD01G1106900), *MdCHS* (MD04G1003300), *MdCHI* (MD07G1186300), *MdF3H* (MD15G1246200), *MdDFR* (MD15G1024100), *MdANS* (MD06G1071600), *MdUFGT* (MD01G1234400), *MdGSTF12* (MD17G1272100), *MdMYB1* (MD09G1278600), *MdMYB9* (MD08G1070700), *MdMYB10* (MD09G1278400), *MdMYB110a* (MD17G1261000), *MdbHLH3* (MD11G1286900), *MdbHLH33* (MD07G1137500), *MdTTG1* (MD14G1031200), *MdActin* (LOC103453508), and *NbActin* (LOC107795436).

## Results

### Aminolevulinic Acid and Eth Increase Anthocyanin Accumulation in Apple Fruits

**FIGURE 1:**
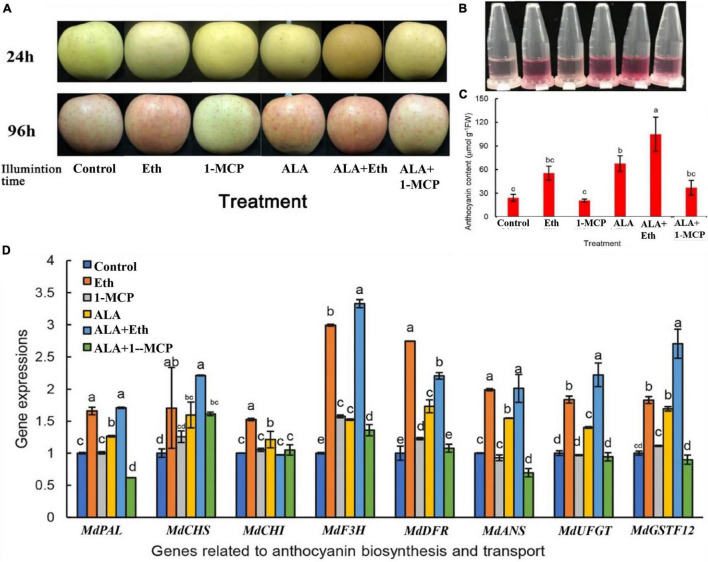
Effects of ALA and ethephon on the coloration of “Gala” apple fruits. **(A)** Coloring comparison of “Gala” apple fruits after ALA, ethylene, and 1-MCP treatments. **(B)** Anthocyanin extracting solutions from the fruit peels. **(C)** Comparison of the anthocyanin content in apple peel. **(D)** The relative expressions of the genes involved in anthocyanin biosynthesis and transport after ALA, ethylene, and 1-MCP treatments. The data are means ± SE of three biological replicates. The same letters above the bars represent no significant differences at *p* = 0.05 (*t*-test).

As shown in Figure 1 both ALA and Eth increased anthocyanin accumulation in apple fruits. When the bagged “Gala” apple fruits that were treated with 300 mg L^–1^ Eth or 200 mg L^–1^ ALA were exposed to light for 4 days, the skins became redder than the control (Figure 1A). Measurements also showed that the anthocyanin content in ALA or Eth treatment were more than double when compared with that of the control (Figures 1B,C). If the fruits were treated by Eth + ALA, the anthocyanins were 4.3 times as high as that of the control, suggesting a synergistic effect occurs between Eth and ALA. 1-MCP itself did not significantly affect coloring and the anthocyanin content of the fruits. However, it inhibited the ALA-induced anthocyanin accumulation. When fruits were treated by ALA after 1-MCP fumigation, the anthocyanin content was about half of that without 1-MCP treatment, suggesting that ALA-induced anthocyanins may be dependent on ethylene signals.

Both ALA and Eth also induced the expressions of genes involved in anthocyanin biosynthesis and transport, including *MdPAL*, *MdCHS*, *MdCHI*, *MdF3H*, *MdANS*, *MdUFGT*, and *MdGSTF12*, while 1-MCP inhibited the upregulation of gene expression induced by ALA (Figure 1D). Thus, it seems that ALA-induced gene expressions are at least partially dependent on ethylene signals. Furthermore, correlation analysis revealed that the anthocyanin content of the peels was all positively correlated with the above gene expressions, although only *MdCHS, MdANS*, *MdUFGT*, and *MdGSTF12* were statistically significant, with correlation coefficients 0.871^∗^, 0.816^∗^, 0.907^∗^, and 0.949^∗∗^, respectively. It seems that the regulatory sites of ALA or Eth may be in the late biosynthetic genes, especially in the transport of anthocyanins, since *MdGSTF12* is important for anthocyanin transport in apple.

### Identification of an Anthocyanin-Regulated Candidate Gene, *MdERF78*

**FIGURE 2:**
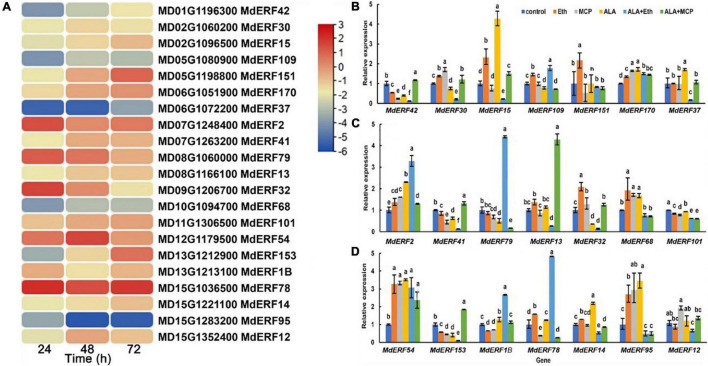
MdERF78 was identified as a candidate regulator involved in ALA-induced anthocyanin accumulation. **(A)** Heat map showing the ERF TFs expression pattern of “Fuji” apple flesh calli under ALA treatment for 24, 48, and 72 h. **(B–D)** The relative expressions of *MdERFs* in “Gala” apple fruits after ALA, ethylene, and 1-MCP treatments. The data are means ± SE of three biological replicates. The same letters above the bars represent no significant differences at *p* = 0.05 (*t*-test).

Ethylene responsive factors (ERF) are an important group of transcription factors (TFs) involved in the ethylene signaling transduction and anthocyanin accumulation. In our previously published transcriptome database of ALA-treated apple calli (*Malus domestica* cv. “Fuji”), a total of 21 differentially expressed ERFs were identified (Zheng et al., 2021). Among them, the expressions of *MdERF78* (MD15G1036500), *MdERF79* (MD08G1060000), and *MdERF2* (MD07G1248400) were increased in all three time-points after ALA treatment based on RNA-seq data (Figure 2A).

To clarify the responses of MdERF gene expressions to ALA, we analyzed the transcript levels of all 21 MdERFs in “Gala” apples treated with Eth, ALA, 1-MCP, ALA + Eth, and ALA + 1-MCP by qRT-PCR, and found that some of MdERFs were upregulated by Eth and ALA but downregulated by 1-MCP (Figures 2B–D). Analysis of the correlation between gene expressions and the anthocyanin content showed that the transcript levels of *MdERF1B* (MD13G1213100), *MdERF2* (MD07G1248400), *MdERF78* (MD15G1036500), and *MdERF79* (MD08G1060000) were significantly correlated, with correlation coefficients 0.853, 0.835, 0.916, and 0.816, respectively (Table 1). The correlation coefficient of *MdERF109* expression with the anthocyanin content was also high up to 0.736; however, it was not significant at *p* = 0.05. Additionally, since the correlation coefficient of *MdEFR78* expression with the anthocyanin content was the highest among the *ERFs*, we analyzed the correlation between *MdERF78* expression and that of the genes involved in anthocyanin biosynthesis and transport, and the results showed that *MdERF78* was significantly correlated with *MdUFGT* and *MdGSTF12*, with coefficients of 0.90 and 0.94, respectively (Supplementary Table 2). Therefore, we considered that *MdERF78* is an important gene regulating ALA-induced anthocyanin biosynthesis, worth further study.

**TABLE 1:** Correlation analysis between the expression of *MdERFs* and the anthocyanin content.

Gene name	Pearson correlation	Gene name	Pearson correlation	Gene name	Pearson correlation
*MdERF12*	–0.760	*MdERF32*	–0.484	*MdERF79*	0.816*
*MdERF13*	–0.255	*MdERF37*	–0.552	*MdERF95*	–0.321
*MdERF14*	–0.181	*MdERF41*	–0.509	*MdERF101*	–0.465
*MdERF15*	–0.058	*MdERF42*	–0.446	*MdERF109*	0.736
*MdERF1B*	0.853*	*MdERF54*	0.345	*MdERF151*	0.191
*MdERF2*	0.835*	*MdERF68*	–0.303	*MdERF153*	–0.437
*MdERF30*	–0.824	*MdERF78*	0.916*	*MdERF170*	0.195

*Represents the correlation significance at p = 0.05.

According to the apple genome annotation, the open reading frame (ORF) region encodes 180 amino acid residues in MdERF78. Phylogenetic analysis showed that MdERF78 is very close to MdERF109 (Supplementary Figure 1A). The conserved domain analysis indicated that MdERF78 contains an AP2 conserved domain at the N-terminus, highly similar to other ERFs from different species (Supplementary Figure 1B). The subcellular localization analysis showed that MdERF78 was located in the nucleus (Supplementary Figure 1C).

### MdERF78 Promotes Anthocyanin Accumulation in Apple Fruits and Calli

**FIGURE 3:**
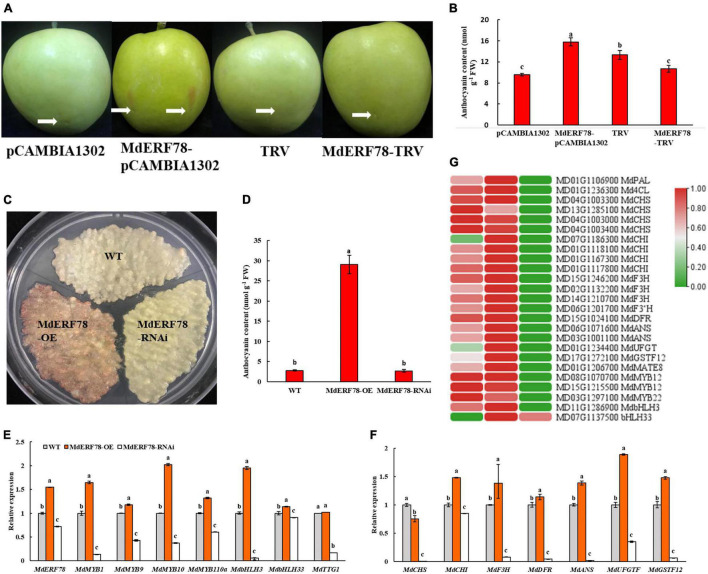
Effects of transient or stable transformed *MdERF78* on fruit coloration and anthocyanin accumulation in apple fruits or calli. **(A)** The anthocyanin accumulation in the skin of “Gala” apple fruits injected by MdERF78-pCAMBIA1302 (overexpression) vector or MdERF78-TRV (TRV1 + TRV2, interference) vector. The white arrows indicate the positions of injection. **(B)** Anthocyanin content in the transiently transformed apple peel. **(C)** Anthocyanin accumulation in the “Orin” apple calli which were stably transformed by MdERF78-OE vector or MdERF78-RNAi vector **(D)** comparison of the anthocyanin content among genotypes in transgenic calli. **(E,F)** The relative expressions of **(E)** transcription factors and **(F)** structural genes associated with anthocyanin biosynthesis and transport in transgenic calli. **(G)** Comparison of expressions of the genes associated with anthocyanin accumulation between the transgenic apple calli and the WT detected by RNA-seq. The heatmap was plotted with log_2_RPKM with zero to one scale method to visually show differences in expression levels. The data are means ± SE of three biological replicates. The same letters above the bars represent no significant differences at *p* = 0.05 (*t*-test).

To elucidate the biological roles of MdERF78 in the regulation of anthocyanin accumulation, an overexpression vector MdERF78-pCAMBIA1302 and a silencing vector MdERF78-TRV were constructed and injected into “Gala” apple fruits, respectively. After 3 days of light exposure, the skin injected with *MdERF78* overexpression became red but the other treatments including empty vectors and MdERF78-TRV did not have any such effect (Figure 3A). Spectrophotometry determination showed that the anthocyanin content in the *MdERF78* overexpression was 64% higher than that of the empty, but when *MdERF78* was silenced by TRV, the content was decreased to the comparable level of empty. It suggests that MdEFR78 can promote anthocyanin accumulation in apple skins (Figure 3B).

When either overexpression (MdERF78-OE) or interference (MdERF78-RNAi) of *MdERF78* was stably transformed into “Orin” apple calli mediated by *Agrobacterium* (Supplementary Figure 2), the result showed that the MdERF78-OE calli accumulated near 10-fold more anthocyanin than the wild-type (WT), while no pigmentation accumulated in MdERF78-RNAi calli after 7-days of illumination (Figures 3C,D). When detected by qRT-PCR, the relative expressions of both regulatory genes (including *MdERF78, MdMYB1*, *MdMYB9*,*MdMYB10*, *MdMYB110a*, *MdbHLH3*, and *bHLH33* except *MdTTG1*) and structural genes (including *MdCHI*, *MdF3H*, *MdDFR*, *MdANS*, *MdUFGT, and MdGST*F12) in the MdERF78-OE were upregulated, but all including *MdTTG1* were downregulated in the MdERF78-RNAi (Figures 3E,F). These results suggest that MdERF78 might be located upstream of regulatory routes, necessary for many gene expressions involved in anthocyanin biosynthesis and transport.

We used RNA-seq technology to elucidate the connection between *MdERF78* and anthocyanin accumulation. When MdERF78-OE and MdERF78-RNAi apple calli were exposed to the strong light for 7 days, it was found that 1,297 DEGs (728 upregulated and 569 downregulated) were detected in the MdERF78-OE, while 2,637 DEGs (1,340 upregulated and 1,297 downregulated) were detected in the MdERF78-RNAi compared with the WT (Supplementary Figure 3). KEGG analysis indicated that the DEGs between WT and MdERF78-OE were mainly fallen in the pathways including phenylpropanoid biosynthesis, photosynthesis, and pentose and glucuronate interconversions. The pathways of the DEGs between WT and MdERF78-RNAi were found mainly associated with flavonoid biosynthesis, phenylpropanoid biosynthesis, and nitrogen metabolism (Supplementary Figure 4). These once again confirmed that *MdERF78* participated in the regulation of anthocyanin biosynthesis. We further analyzed the expressions of genes related with anthocyanin biosynthesis, transport, and regulation, and the results revealed that most of the genes listed in Figure 3G were up-regulated in expression in the MdERF78-OE but downregulated in the MdERF78-RNAi calli, suggesting that their expressions were dependent on MdERF78. For example, *MdF3Hs* (MD15G1246200, MD02G1132200, and MD14G1210700), *MdANSs* (MD06G1071600 and MD03G1001100), *MdUFGT* (MD01G1234400), *MdGSTF12* (MD17G1272100), and *MdMATE8* (MD01G1206700) were upregulated in the overexpressing *MdERF78* but downregulated in the MdERF78-RNAi calli. All these support that *MdERF78* activates the transcription of anthocyanin-related genes in apples to promote anthocyanin accumulation.

### MdERF78 Plays a Positive Role in Aminolevulinic Acid-Promoted Anthocyanin Accumulation

**FIGURE 4:**
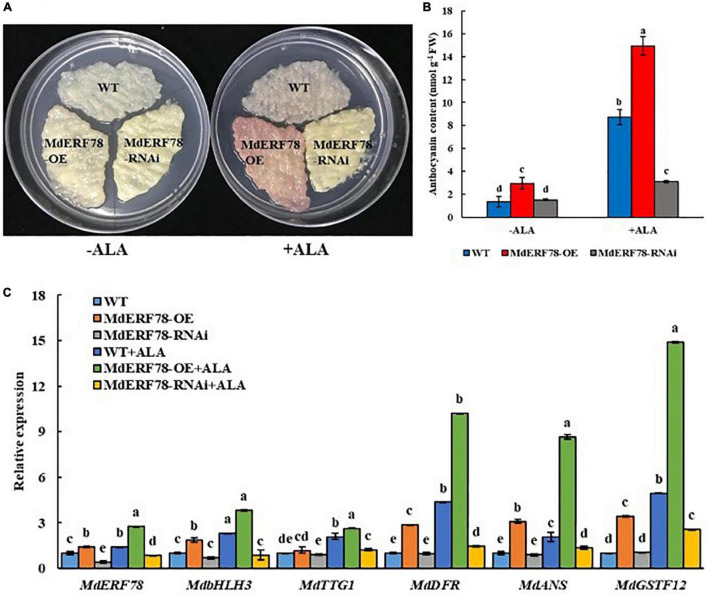
The effects of MdERF78 in ALA-promoted anthocyanin accumulation. **(A)** Anthocyanin accumulation of transgenic apple calli supplement with ALA or without ALA. **(B)** Comparison of the anthocyanin content of apple calli supplemented with or without ALA. **(C)** qRT-PCR analyzed the gene expression related to anthocyanin accumulation in transgenic apple calli supplemented with or without ALA. Values are means ± SE of three biological replicates. The same letters above the bars represent no significant differences at *p* = 0.05 (*t*-test).

Since *MdERF78* was upregulated by ALA and contributed to anthocyanin biosynthesis, we planned to clarify if it was involved in ALA-regulated anthocyanin accumulation. Therefore, we used ALA to treat the MdERF78-OE and MdERF78-RNAi apple calli. The results showed that the anthocyanin content in the MdERF78-OE calli cultured on the medium without ALA after 3 days of light exposure was more than twofold as high as that of the WT, while that was increased by 7 times when ALA was added. It suggests that ALA promoted more anthocyanin accumulation in the *MdERF78* overexpression cells. However, the interference of MdERF78 significantly impaired ALA-induced anthocyanin accumulation (Figures 4A,B), suggesting that ALA-induced anthocyanin accumulation was dependent on MdERF78. Furthermore, qRT-PCR analysis revealed that *MdERF78* overexpression upregulated the gene expressions, such as *MdbHLH3*, *MdTTG1, MdDFR*, *MdANS, MdGSTF12*, as well as *MdERF78* itself, while MdERF78-RNAi downregulated all the gene expressions (Figure 4C). It can be deduced that *MdERF78*, acting at the upper stream of signaling routes, beyond the regulatory genes and structural genes, plays a positive role in the ALA-promoted anthocyanin accumulation.

### MdERF78 Activates *MdF3H* and *MdANS* Transcription by Directly Binding to Their Promoter

**FIGURE 5:**
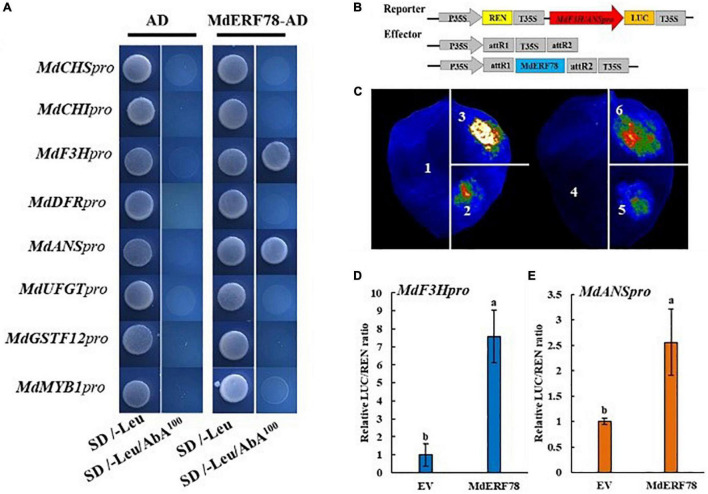
Effect of MdERF78 on the promoters of anthocyanin-related genes. **(A)** Y1H analysis of the interaction of MdERF78 and the promoters related with anthocyanin biosynthesis, transport and regulation. **(B)** Schematic diagrams of effector and reporter vectors for the dual-luciferase assays. **(C)** Luciferase complementation imaging assays showing that MdERF78 activates *MdF3Hpro* and *MdANSpro*. 1. pGreenII 62-SK + pGreenII 0800-LUC, 2. pGreenII 62-SK + *MdF3Hpro*-pGreenII 0800-LUC, 3. MdERF78-pGreenII 62-SK + *MdF3Hpro*-pGreenII 0800-LUC, 4. pGreenII 62-SK + pGreenII 0800-LUC, 5. pGreenII 62-SK + *MdANSpro*-pGreenII 0800-LUC, 6. MdERF78-pGreenII 62-SK + *MdANS*pro-pGreenII 0800-LUC. **(D,E)** Quantitative analysis of **(D)** *MdF3H* promoter and **(E)** *MdANS* promoter luminescence intensity increased by MdERF78. Values are means ± SE of three biological replicates. The same letters above the bars represent no significant differences at *p* = 0.05 (*t*-test).

Experiments were carried out to test the possibility that *MdERF78* activates gene expressions related with anthocyanin biosynthesis and transport. In a Y1H assay, the promoters of *MdCHI*, *MdCHS*, *MdF3H*, *MdDFR*, *MdANS*, *MdUFGT*, *MdGSTF12*, and *MdMYB1* were separately fused into the pAbAi vector, whereas the CDS of *MdERF78* was inserted into the pGADT7 vector. When the fused pAbAi vectors were co-expressed with MdERF78-AD, only *MdANSpro*-pAbAi or *MdF3Hpro*-pAbAi could grow on an SD/-Leu/AbA^100^ plate, while the negative control and other combinations did not grow (Figure 5A). These results suggest that MdERF78 may directly bind to the promoters of *MdF3H* and *MdANS* but not the others.

To determine whether MdERF78 could activate the promoters of *MdANS* and *MdF3H*, a dual-luciferase assay was carried out. The effector plasmid contained the CDS of *MdERF78* driven by the *35S* promoter, and the *MdF3H* and *MdANS* promoter fragment (2000bp) fused into a luciferase reporter vector (Figure 5B). The effector plasmids and the reporter plasmids mediated by *Agrobacterium* were injected into tobacco leaves for co-expression. Luciferase complementation imaging assays showed that compared with an empty vector, the overexpression of *MdERF78* significantly increased the luminescence intensity (Figure 5C). Consistent with luminescence intensity phenotype, MdERF78 increased the *MdF3H* and *MdANS* promoter activities by 7-fold and 2.5-fold, respectively compared with that of the control vector (Figures 5D,E). Collectively, these results support that MdERF78 activates *MdF3H* and *MdANS* by directly binding to their promoters.

### MdERF78 Interacts With MdMYB1 but Not MdbHLH3

**FIGURE 6:**
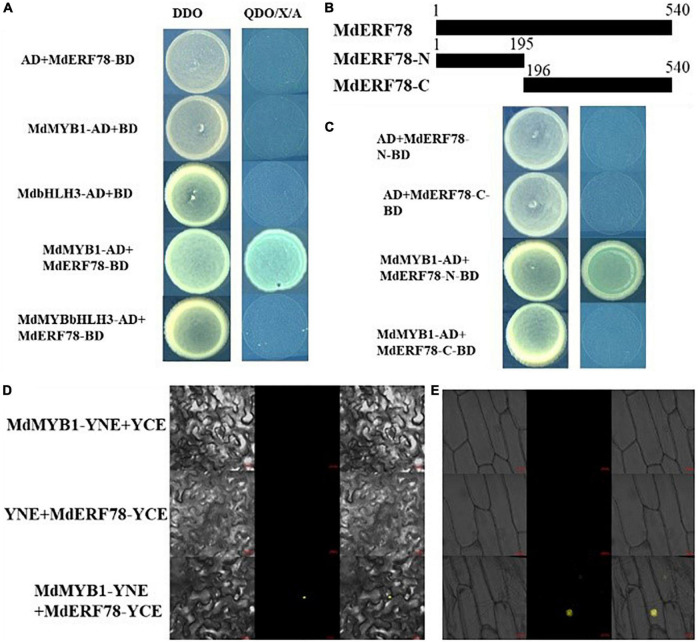
MdERF78 interacted with MdMYB1 but not MdbHLH3. **(A)** Interaction between MdERF78 and MdMYB1 but not MdbHLH3 in Y2H assays. DDO, double-dropout medium; QDO/X/A, quadruple-dropout medium added X-α-gal and AbA. **(B)** Schematic diagrams of different parts of MdERF78. **(C)** Interaction of MdMYB1 with different parts of MdERF78 **(D,E)** BiFC assays demonstrated MdMYB1 interacts with MdERF78 in the nuclei of **(D)** tobacco leaves and **(E)** epidermal cells of onions. Scale bars = 20 μm.

Previous studies have demonstrated that MYB1 (Takos et al., 2006; An et al., 2020a) and bHLH3 (Xie et al., 2012) play crucial roles in the regulation of anthocyanin biosynthesis. To verify whether MdERF78 interacts with those two TFs, a Y2H assay was performed to explore the relationship between MdERF78 and MdMYB1 or bHLH3. Results showed that on co-expressed MdERF78-BD + MdMYB1-AD, the yeast strain grew normally and turned blue on the selection medium, whereas MdERF78-BD + MdbHLH3-AD did not grow in the same medium (Figure 6A). The is indicated that MdERF78 can interact with MdMYB1 but not MdbHLH3. Moreover, MdERF78 was divided into an N-terminal domain MdERF78N^1–65^ and a C terminal domain MdERF78C^66–180^, and was then inserted into the pGBKT7 vectors, respectively (Figure 6B). The co-transformed yeast strains containing MdMYB1-AD and MdERF78-N^1–65^-BD grew normally and turned blue on the selection medium, in contrast to the lack of growth for the other strains (Figure 6C). The results suggest that MdMYB1 interacts with the N terminal of MdERF78 rather than the C terminal in yeast cells.

Additionally, BiFC assays were carried out with tobacco leaves and onion epidermal cells to verify the interaction between MdERF78 and MdMYB1. The result showed that the yellow fluorescence signal could be observed in the nuclei of tobacco leaves and onion skin cells when co-transformed with MdMYB1-YFPC and MdERF78-YFPN, but there was no fluorescent signal in the controls and other combinations (Figures 6D,E). These results suggest that MdERF78 interacts with MdMYB1 in the nucleus.

### Interaction of MdMYB1 With MdERF78 Enhances Its Transcriptional Activity

**FIGURE 7:**
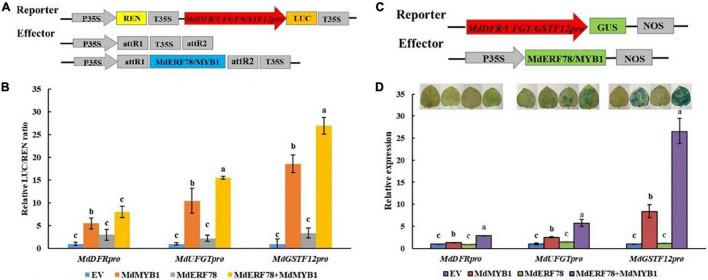
Effect of MdERF78 on the MdMYB1 target genes including *MdDFR*, *MdUFGT* and *MdGSTF12*. **(A)** Schematic diagrams of effector and reporter vector for the dual-luciferase assays. **(B)** Quantitative analysis of *MdDFR*, *MdUFGT* and *MdGSTF12* promoter luminescence intensity increased by MdERF78, MdMYB1 and MdERF78 + MdMYB1. **(C)** Schematic diagrams of effector and reporter vector for the GUS assays. **(D)** GUS staining on tobacco leaves and *GUS* expression by RT-qPCR revealed MdERF78 enhance the transcriptional regulatory effect of MdMYB1 to *MdDFR*, *MdUFGT* and *MdGSTF12*. The data are means ± SE of three biological replicates. The same letters above the bars represent no significant differences at *p* = 0.05 (*t*-test).

To investigate whether the MdERF78-MdMYB1 interaction affects the transcription activity on the target gene promoters, the dual luciferase assays were performed (Figure 7A). As shown in Figure 7B, overexpression of *MdMYB1* alone was able to increase the LUC/REN ratio, whereas MdERF78 itself was not. When *MdERF78* and *MdMYB1* were co-expressed, the LUC/REN was much higher than *MdMYB1* overexpression solo. The results suggest that MdERF78 enhances MdMYB1 transcriptional activity on its target genes, such as *MdDFR*, *MdUFGT*, and *MdGSTF12*. However, MdERF78 itself has no transcription activity on the genes.

To further verify the results, we performed transient GUS activity assays in tobacco leaves (Figure 7C). It can be seen that *MdERF78* itself did not promote leaf GUS staining, but *MdMYB1* overexpression obviously made tobacco leaves blue. When *MdERF78* was co-transformed with *MdMYB1*, the tobacco became much bluer than *MdMYB1* transformation solo. Variation of leaf staining was also reflected in *GUS* gene expression (Figure 7D). These indicate that MdERF78 itself does not promote the gene transcriptions, but the MdERF78-MdMYB1 interaction enhances the transcriptional activity of MdMYB1 to the promoters of *MdDFR*, *MdUFGT*, and *MdGSTF12*.

## Discussion

### MdERF78 Plays an Essentially Positive Role in the Response of Aminolevulinic Acid to Anthocyanin Accumulation

As a plant-specific transcription factors, the AP2/ERF family is involved in the plant life process. They not only participate in the primary metabolism of plants but are also involved in secondary metabolism, such as anthocyanin biosynthesis and transport (Mizoi et al., 2012; Feng et al., 2020). In *Arabidopsis*, AtERF4 and AtERF8 are reported to be involved in light-induced anthocyanin biosynthesis (Koyama and Sato, 2018). In pear, the transient transformation of *PyERF3* with *PyMYB114* and *PybHLH3* in tobacco leaves, strawberry, and pear fruits enhanced anthocyanin accumulation (Yao et al., 2017). In apple, overexpression of *MdERF1B* promoted anthocyanin accumulation in apple calli (Zhang et al., 2018). In a previous study, we observed that more than twenty *MdERFs* were differentially expressed after ALA treatment in RNA-seq data (Zheng et al., 2021). Therefore, we suspected that at least some of them might be contributed to ALA-promoted anthocyanin accumulation. In the present work, we firstly used ethephon, 1-MCP, and ALA to treat “Gala” apple fruits, and found that ethylene seemed involved in fruit coloration and anthocyanin accumulation (Figure 1A). Comprehensive analyses show that *MdERF78* may be the most possible candidate gene responsible for ALA-induced anthocyanin accumulation. Its expression was induced by ALA in “Fuji” apple calli based on RNA-seq data (Figure 2A), as well as by ALA + Eth in “Gala” apple fruits detected by qRT-PCR (Figures 2B–D), with the highest correlation coefficient with the anthocyanin content among all the *MdERFs* (Table 1). The phylogenetic analysis showed that MdERF78 and MdERF109 were clustered into one sub-group (Supplementary Figure 1A), and the latter was reported to be involved in light-induced anthocyanin biosynthesis (Ma et al., 2021). Thus, MdERF78 may share a similar regulatory function with MdERF109 in anthocyanin biosynthesis.

As expected, *MdERF78* overexpression promoted anthocyanin accumulation, while *MdERF78* silence blocked the accumulation (Figures 3A–D). Consistent with changes in anthocyanin content, the genes related to anthocyanin biosynthetic and transport pathways were up- or down-regulated in the overexpression or interference apple calli, respectively (Figures 3E–G). Therefore, we propose that *MdERF78* expression is necessary for anthocyanin accumulation in apples.

5-Aminolevulinic acid has been known to be able to promote anthocyanin accumulation in many species of fruits (Wang et al., 2004; Guo et al., 2013; Ye et al., 2017; Zhang et al., 2021), and the new natural plant growth regulator can be applied in high-quality fruit production (Xie et al., 2013; Wu et al., 2019). Previous studies have proposed that the other TFs such as MdMYB9, MdMYB10 (Zheng et al., 2021), and MdMADS1 (Feng et al., 2016) are also necessary for ALA to induce anthocyanin accumulation in apples. However, whether AP2/ERF TFs are involved in ALA-promoted anthocyanin accumulation was not clear before. In this work, anthocyanin accumulation in MdERF78-OE calli was further enhanced by treatment with ALA. However, *MdERF78* interference significantly inhibited ALA-induced anthocyanin accumulation in apple calli (Figures 4A,B). These suggest that MdERF78 is essentially a positive regulator in ALA-promoted anthocyanin accumulation.

### MdERF78 Positively Regulates Anthocyanin Accumulation at Multiple Levels

It is known that ERFs regulate anthocyanin accumulation by directly binding to the promoters of anthocyanin-related genes. In strawberry, an ERF has been identified to bind to the regulatory gene *FaMYB10* and structural genes like *CHS*, *F3H*, *DFR*, and *GT1* promoters (Zhang et al., 2020b). In apple, MdERF1B binds to the promoters of *MdMYB1*, *MdMYB9*, and *MdMYB11*, which were known to be important regulatory genes in anthocyanin accumulation (Zhang et al., 2018). Similarly, MdERF3 can indirectly activate the transcription of *MdMYB1* to promote anthocyanin biosynthesis (An et al., 2018a), while, MdERF109 binds to the promoters of *MdCHS*, *MdUFGT*, and *MdbHLH3* (Ma et al., 2021). In gene promoters, DREB (Stockinger et al., 1997; Liu et al., 1998; Sakuma et al., 2002), RAV (Kagaya et al., 1999), and GCC-box (Fujimoto et al., 2000) are often the binding motifs of ERFs. In apple, we analyzed the promoter sequences of the structural genes as well as the regulator genes related to anthocyanin biosynthesis and transport, and found that all these target motifs existed in the promoters of the genes (Supplementary Figure 5). Therefore, the fact that MdERF78 binds to the sites of promoters is very possible. Here, we confirmed that MdERF78 activates the expressions of anthocyanin biosynthetic genes, *MdF3H* and *MdANS*, by directly binding to their promoters (Figure 5). Since *MdF3H* and *MdANS* are the necessary structural genes of the anthocyanin biosynthesis pathway (Honda et al., 2002), the activation of gene expressions by MdERF78 may facilitate the anthocyanin accumulation. On the other hand, MdERF78 did not bind to the promoters of the other genes such as *MdCHS*, *MdCHI*, *MdDFR*, *MdUFGT*, *MdGSTF12*, and *MdMYB1* in yeast cells (Figure 5A), implying no direct activation of the gene expressions.

Furthermore, we found that MdERF78 interacted with MdMYB1 (Figure 6), which is consistent with previous studies that MdERF1B interacts with MdMYB9 and MdMYB11 (Zhang et al., 2018), and MdERF38 interacts with MdMYB1 to promote anthocyanin accumulation (An et al., 2020b). It seems that ERF TFs can interact with MYB TFs and then co-regulate gene expressions for anthocyanin accumulation. However, MdERF109, the most common homolog to MdERF78 in the phylogenetic tree (Supplementary Figure 1A), did not interact with MdMYB1, but directly activated the anthocyanin-related gene expressions (Ma et al., 2021). On the other hand, bHLHs, another core component of the MBW complex, has been suggested to interact with MYBs but not ERFs in sweet potato (Ning et al., 2021) or pear (Yao et al., 2017), which was also found in the present study. From Figure 6A, there was no interaction between MdERF78 and MdbHLH3. These results suggest that different ERF proteins share similar functions in anthocyanin accumulation, but through different regulatory mechanisms. They may directly promote or interact with MYB, but not with bHLH, and indirectly promote the structural gene expressions, leading to anthocyanin biosynthesis and transport.

MdMYB1 is an important positive regulator in anthocyanin accumulation. It can directly bind to and activate the downstream structural genes of the anthocyanin biosynthesis pathway (Takos et al., 2006). Additionally, it has been reported that the regulatory function of MdMYB1 can be enhanced by its interaction with MdERF38 (An et al., 2020b), MdbZIP44 (An et al., 2018b), or MdWRKY40 (Zhang et al., 2019). In the present study, we demonstrated that MdERF78 enhanced the MdMYB1 transcriptional regulation to promote its downstream target gene expressions, including *MdDFR*, *MdUFGT*, and *MdGSTF12* (Figures 7B,D). It was valuable to note that MdERF38, which was involved in drought-induced anthocyanin accumulation, shares a similar anthocyanin regulatory mechanism with MdERF78. Both of them could enhance the transcriptional activity of MdMYB1 to its target genes *MdDFR* and *MdUFGT* by interacting with MdMYB1 (An et al., 2020b). Thus, ALA-induced MdERF78 may interact with MdMYB1 to promote the expressions of the target genes of anthocyanin accumulation.

### Crosstalk Between Aminolevulinic Acid and Ethylene

Anthocyanin synthesis is modulated by a complex regulatory network of feedback and crosstalk among multiple phytohormone signaling pathways (Jaakola, 2013). Interestingly, ALA not only has hormonal properties but also interacts with a variety of hormones to regulate plant growth and development (Bindu and Vivekanandan, 1998; An et al., 2016; Liu et al., 2019). Exogenous ALA treatment can increase endogenous JA content in tomatoes to improve plant cold tolerance (Liu et al., 2019). ALA can also promote the primary root growth in *Arabidopsis* by mediating auxin polar transport (An et al., 2019b). Treatment with 24-epibrassinolide (24-EBL) promoted ALA-induced anthocyanin accumulation in “Fuji” apple calli, while brassinazole (Brz), an inhibitor of brassinolide biosynthesis suppressed ALA-induced coloration. In the guard cells of apple, ethylene is involved in the regulation of stomatal movement by ALA-ABA/dark (Hu et al., 2020). Therefore, the plant hormones may be involved in ALA-induced anthocyanin metabolism in apples (Zheng et al., 2018). In the present study, we once again found that treatments with ALA, ethephon alone, or together promoted anthocyanin accumulation, which was consistent with previous studies. Pretreatment of apples with the ethylene inhibitor 1-MCP reduced ALA-induced anthocyanin accumulation (Figures 1A,B). MdERF78, which is responsive to ALA and ethylene, plays a positive role in regulating anthocyanin accumulation. It suggests that a crosstalk between ALA and ethylene might occur in mediating the anthocyanin biosynthesis process in apples. Feng et al. (2016) reported that ACO1, a key enzyme in the ethylene biosynthetic route, is responsive to ALA treatment at protein and mRNA levels in apples. Rupasinghe and Gladon (1996) even suggested that ALA might be metabolized to ethylene during tomato fruit ripening. However, in the report of Wang et al. (2004), they did not find ALA treatment promoting fruit senescence. Song et al. (2008) found that ALA treatment before harvest would postpone spinach senescence. These suggest that ALA treatment has no relation to plant senescence. It should be pointed out that the relationship between ALA and ethylene in apple should be further researched. In the current study, we did not observe MdERF78 directly binding to the promoters of *MdACO1* and *MdACS1* in the Y1H assay (Supplementary Figure 6). It suggests that MdERF78 does not promote the ethylene biosynthetic gene expressions. Therefore, whether additional mechanisms exist requires further study.

## Conclusion

**FIGURE 8:**
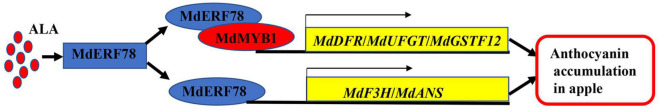
A proposed model of the functioning of MdERF78 in ALAinduced anthocyanin accumulation in apples.

In this study, we demonstrated that MdERF78 promotes anthocyanin accumulation, and it is essential for ALA-induced anthocyanin accumulation. Based on these results, we develop a model for ALA-induced anthocyanin accumulation in apples (Figure 8). *MdERF78*, which is induced by ALA, positively regulates anthocyanin accumulation in two ways. On the one hand, MdERF78 enhances the transcriptional capacity of MdMYB1 to *MdDFR*, *MdUFGT*, and *MdGSTF12* by interacting with MdMYB1. On the other hand, MdERF78 activates the expression of *MdF3H* and *MdANS* by binding to their promoters. Overall, our findings shed new light on the mechanisms of ALA-induced anthocyanin accumulation in apples.

## Data Availability Statement

The datasets presented in this study can be found in online repositories. The names of the repository/repositories and accession number(s) can be found below: NCBI BioProject - PRJNA828375.

## Author Contributions

XF and LW conceived and designed the experiments, wrote, and revise the manuscript. XF and LZ conducted the experiment and analyzed the results. All authors contributed to the article and approved the submitted version.

## Conflict of Interest

The authors declare that the research was conducted in the absence of any commercial or financial relationships that could be construed as a potential conflict of interest.

## Publisher’s Note

All claims expressed in this article are solely those of the authors and do not necessarily represent those of their affiliated organizations, or those of the publisher, the editors and the reviewers. Any product that may be evaluated in this article, or claim that may be made by its manufacturer, is not guaranteed or endorsed by the publisher.

## Supplementary Material

Supplementary Figure 1 Analysis of MdERF78 characteristics. **(A)** Phylogenetic tree analysis of MdERF78 and other anthocyanin- related ERFs from other species. The red triangle shows MdERF78. **(B)** Sequence alignment of MdERF78 with other ERF sequences. AP2 domain was indicated by a red horizontal line **(C)** subcellular localization of MdERF78 in *Nicotiana tabacum* leaves. Bars = 40 μm.

Supplementary Figure 2 Transgenic apple calli were confirmed by PCR amplification (35S and MdERF78 R primers for PCR). M, marker. WT: wild-type; MdERF78-OE, MdERF78-overexpression line; MdERF78-RNAi, MdERF78 interference line.

Supplementary Figure 3 Numbers of DEGs compared between MdERF78-OE and WT, MdERF78-RNAi and WT. DEGs are shown in red (up-regulated) and green (down-regulated).

Supplementary Figure 4 KEGG enrichment analysis of MdERF78-OE transcriptome. The number of DEGs was represented by the size of the points, and the q-value ranges were represented by the colors of the points.

Supplementary Figure 5 C is elements analysis on promoters of the anthocyanin related genes. ERF binding sites including RAV, DREB and GCC are indicated in the promoters of anthocyanin-related genes.

Supplementary Figure 6 Y1H analysis of the interaction between MdERF78 and *MdACO1* or *MdACS1* promoter.
