# Supplementary figures and images for: The Transcription Factor MdERF78 Is Involved in ALA-Induced Anthocyanin Accumulation in Apples
Source: Front Plant Sci. 2022 Jun 2;13:915197. doi: 10.3389/fpls.2022.915197 (PMC9201628; doi:10.3389/fpls.2022.915197)

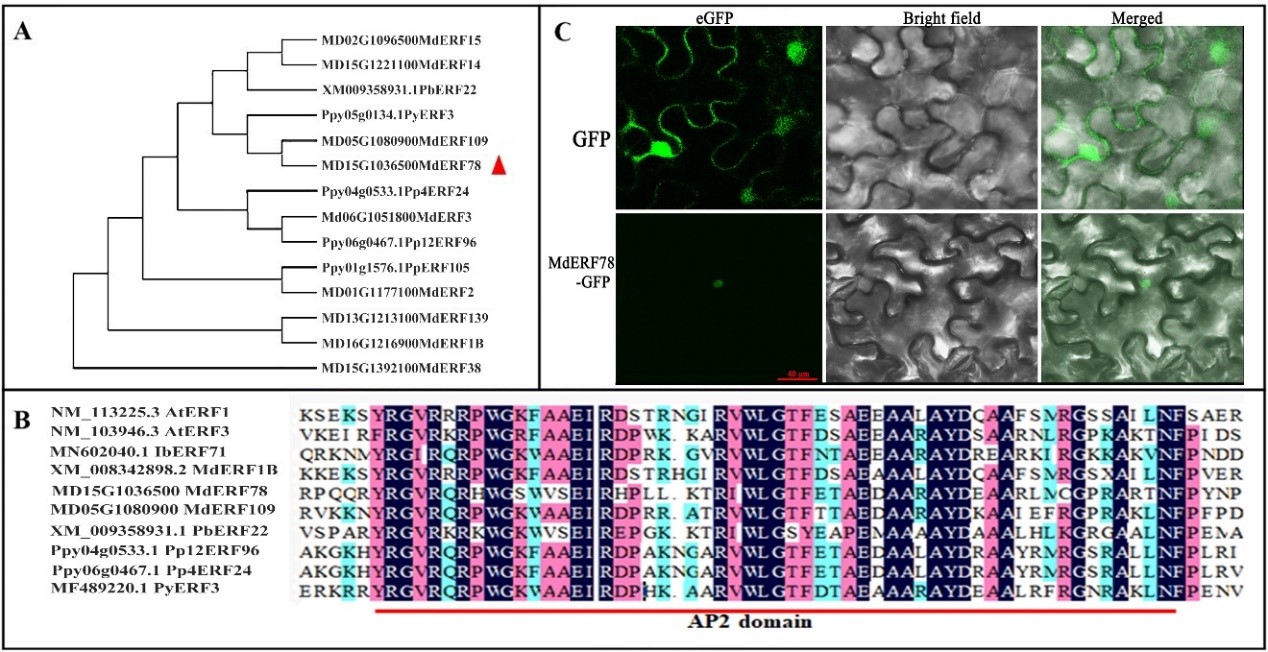

Supplement: Supplementary Figure 1 — Analysis of MdERF78 characteristics. (A) Phylogenetic tree analysis of MdERF78 and other anthocyanin- related ERFs from other species. The red triangle shows MdERF78. (B) Sequence alignment of MdERF78 with other ERF sequences. AP2 domain was indicated by a red horizontal line (C) subcellular localization of MdERF78 in Nicotiana tabacum leaves. Bars = 40 μm. [file Image_1.JPEG]

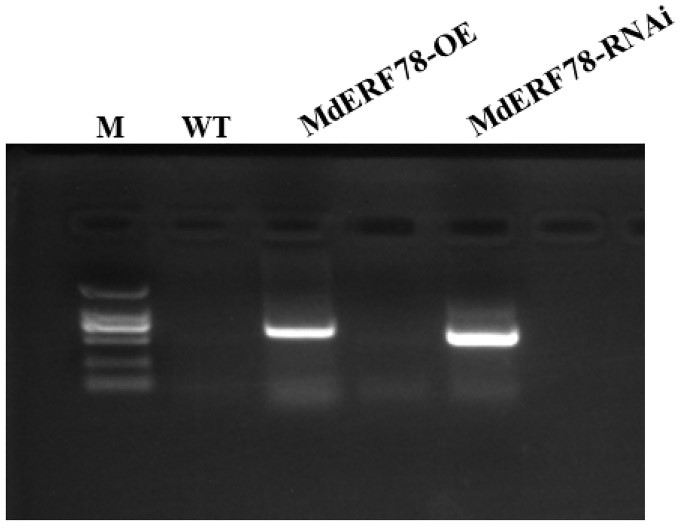

Supplement: Supplementary Figure 2 — Transgenic apple calli were confirmed by PCR amplification (35S and MdERF78 R primers for PCR). M, marker. WT: wild-type; MdERF78-OE, MdERF78-overexpression line; MdERF78-RNAi, MdERF78 interference line. [file Image_2.JPEG]

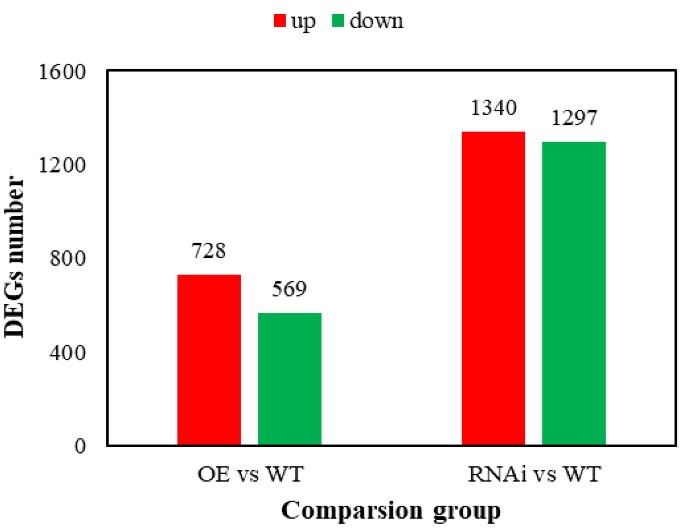

Supplement: Supplementary Figure 3 — Numbers of DEGs compared between MdERF78-OE and WT, MdERF78-RNAi and WT. DEGs are shown in red (up-regulated) and green (down-regulated). [file Image_3.JPEG]

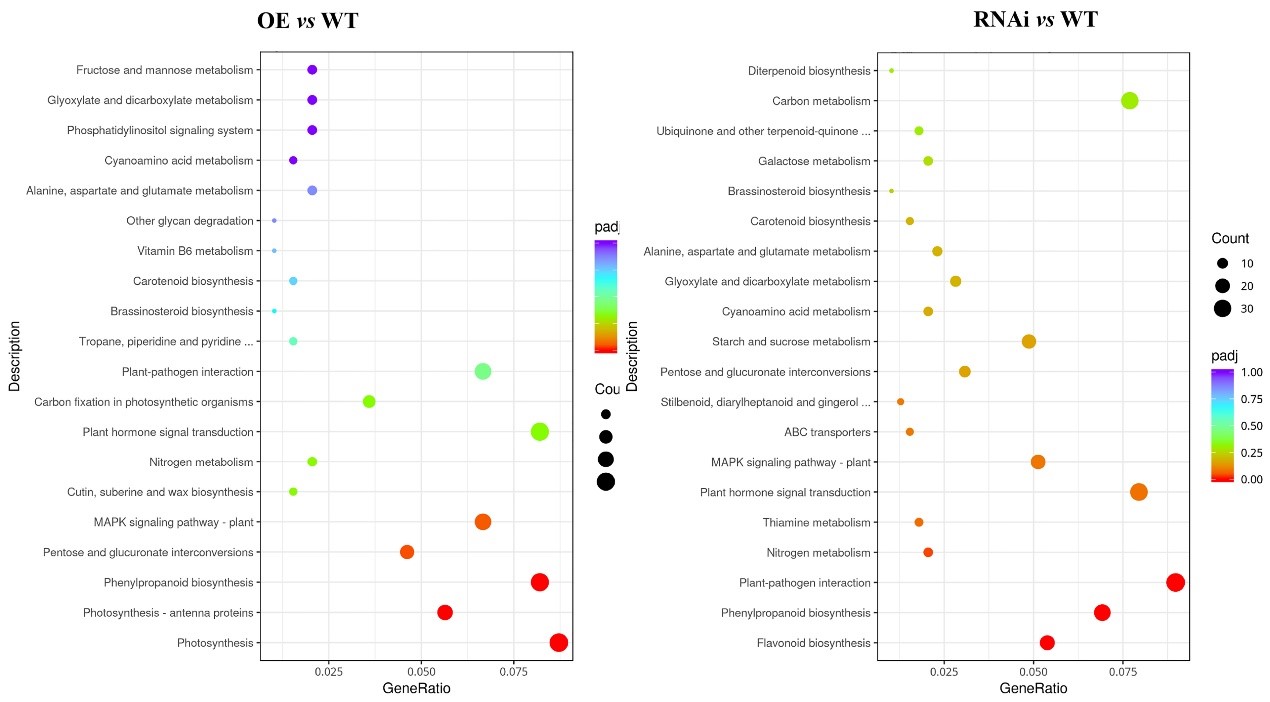

Supplement: Supplementary Figure 4 — KEGG enrichment analysis of MdERF78-OE transcriptome. The number of DEGs was represented by the size of the points, and the q-value ranges were represented by the colors of the points. [file Image_4.JPEG]

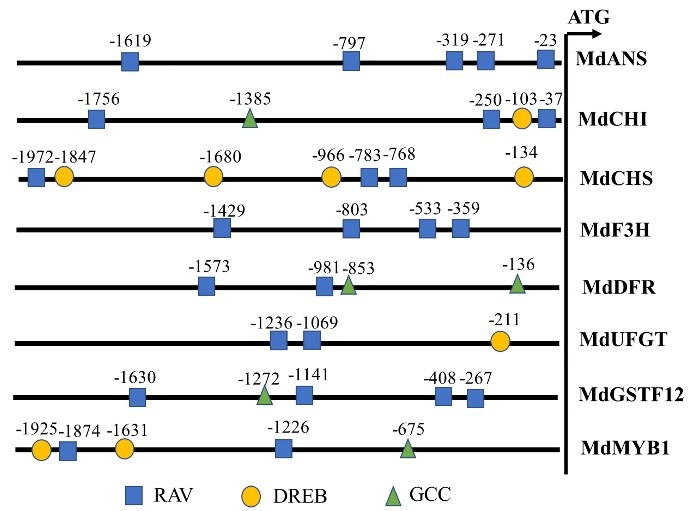

Supplement: Supplementary Figure 5 — C is elements analysis on promoters of the anthocyanin related genes. ERF binding sites including RAV, DREB and GCC are indicated in the promoters of anthocyanin-related genes. [file Image_5.JPEG]

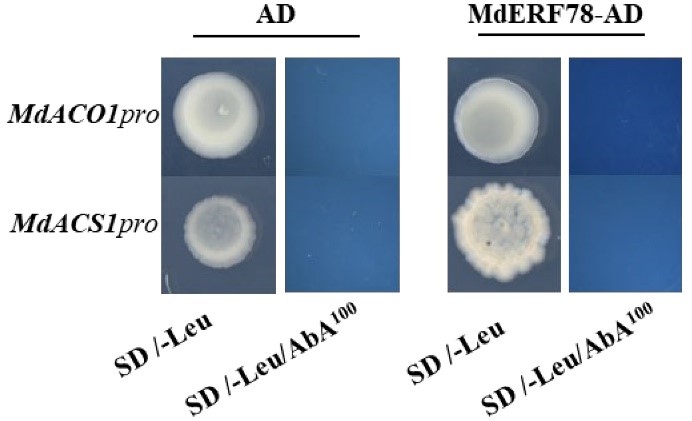

Supplement: Supplementary Figure 6 — Y1H analysis of the interaction between MdERF78 and MdACO1 or MdACS1 promoter. [file Image_6.JPEG]
