# Supplementary material for: The Transcription Factor MdERF78 Is Involved in ALA-Induced Anthocyanin Accumulation in Apples
Source: Front Plant Sci. 2022 Jun 2;13:915197. doi: 10.3389/fpls.2022.915197 (PMC9201628; doi:10.3389/fpls.2022.915197)
Supplement: Supplementary file 8 [file Table_2.docx]

**Supplementary Table 2. Correlation analysis between the expression of *MdERF78* and anthocyanin biosynthesis, transport genes**

| **Correlation** | ***MdPAL*** | ***MdCHS*** | ***MdCHI*** | ***MdF3H*** | ***MdDFR*** | ***MdANS*** | ***MdUFGT*** | ***MdGSTF12*** |
| --- | --- | --- | --- | --- | --- | --- | --- | --- |
| *MdERF78* | 0.78 | 0.78 | -0.15 | 0.80 | 0.58 | 0.77 | 0.90* | 0.94** |
| *MdPAL* |  | 0.62 | 0.44 | 0.87* | 0.91* | 0.98** | 0.93** | 0.90* |
| *MdCHS* |  |  | 0.12 | 0.83* | 0.68 | 0.70 | 0.84* | 0.85* |
| *MdCHI* |  |  |  | 0.36 | 0.71 | 0.50 | 0.30 | 0.14 |
| *MdF3H* |  |  |  |  | 0.89* | 0.87* | 0.94** | 0.88* |
| *MdDFR* |  |  |  |  |  | 0.94** | 0.88* | 0.78 |
| *MdANS* |  |  |  |  |  |  | 0.95** | 0.91* |
| *MdUFGT* |  |  |  |  |  |  |  | 0.97** |
